# Evaluation of the sensory environment in a large tertiary ICU

**DOI:** 10.1186/s13054-023-04744-8

**Published:** 2023-11-27

**Authors:** Oystein Tronstad, Dylan Flaws, Sue Patterson, Robert Holdsworth, Veronica Garcia-Hansen, Francisca Rodriguez Leonard, Ruth Ong, Stephanie Yerkovich, John F. Fraser

**Affiliations:** 1 Critical Care Research Group, Level 3 Clinical Sciences Building, The Prince Charles Hospital, Rode Road, Chermside, QLD 4032 Australia; 2 Faculty of Medicine, University of Queensland, Brisbane, Australia; 3 Physiotherapy Department, The Prince Charles Hospital, Brisbane, Australia; 4 Department of Mental Health, Metro North Mental Health, Caboolture Hospital, Caboolture, Australia; 5 School of Clinical Sciences, Queensland University of Technology, Brisbane, Australia; 6 School of Dentistry, University of Queensland, Brisbane, Australia; 7 School of Architecture and Built Environment, Faculty of Engineering, Queensland University of Technology, Brisbane, Australia; 8 Menzies School of Health Research and Faculty of Health, Queensland University of Technology, Brisbane, Australia

**Keywords:** Acoustics, ICU environment, ICU redesign, Light, Noise, Sound

## Abstract

**Background:**

ICU survival is improving. However, many patients leave ICU with ongoing cognitive, physical, and/or psychological impairments and reduced quality of life. Many of the reasons for these ongoing problems are unmodifiable; however, some are linked with the ICU environment. Suboptimal lighting and excessive noise contribute to a loss of circadian rhythms and sleep disruptions, leading to increased mortality and morbidity. Despite long-standing awareness of these problems, meaningful ICU redesign is yet to be realised, and the ‘ideal’ ICU design is likely to be unique to local context and patient cohorts. To inform the co-design of an improved ICU environment, this study completed a detailed evaluation of the ICU environment, focussing on acoustics, sound, and light.

**Methods:**

This was an observational study of the lighting and acoustic environment using sensors and formal evaluations. Selected bedspaces, chosen to represent different types of bedspaces in the ICU, were monitored during prolonged study periods. Data were analysed descriptively using Microsoft Excel.

**Results:**

Two of the three monitored bedspaces showed a limited difference in lighting levels across the day, with average daytime light intensity not exceeding 300 Lux. In bedspaces with a window, the spectral power distribution (but not intensity) of the light was similar to natural light when all ceiling lights were off. However, when the ceiling lights were on, the spectral power distribution was similar between bedspaces with and without windows. Average sound levels in the study bedspaces were 63.75, 56.80, and 59.71 dBA, with the single room being noisier than the two open-plan bedspaces. There were multiple occasions of peak sound levels > 80 dBA recorded, with the maximum sound level recorded being > 105 dBA. We recorded one new monitor or ventilator alarm commencing every 69 s in each bedspace, with only 5% of alarms actioned. Acoustic testing showed poor sound absorption and blocking.

**Conclusions:**

This study corroborates other studies confirming that the lighting and acoustic environments in the study ICU were suboptimal, potentially contributing to adverse patient outcomes. This manuscript discusses potential solutions to identified problems. Future studies are required to evaluate whether an optimised ICU environment positively impacts patient outcomes.

**Supplementary Information:**

The online version contains supplementary material available at 10.1186/s13054-023-04744-8.

## Introduction

Annually, 13–20 million people are admitted to intensive care units (ICUs) worldwide [1]; associated personal, social, and economic costs are immense. Most patients survive ICU; however, many experience cognitive, physical, and/or psychological impairments and reduced quality of life.

Suboptimal recovery is related to patient (non-modifiable) characteristics, including type of admission (emergent/elective), illness severity, age, gender, and co-morbid physical and mental disorders. Emerging evidence, however, implicates features of the traditional ICU environment in adverse outcomes and suggests environmental modification may improve recovery. Light and noise are identified as particularly problematic, affecting patients and staff [2–8].

ICUs are typically artificially lit with limited patient access to natural lighting. Electrical lighting used typically provides constant low-level illumination that does not mimic the intensity, duration, spectrum, or timing needed to entrain circadian rhythms, impeding patient recovery [9, 10]. Poor lighting and lack of natural light can also contribute to discomfort, stress, and poor health outcomes for staff, and are linked with errors and absenteeism [11–15].

Sound levels in ICU, already higher than international guidelines recommend, are increasing [2, 16–18]. Noise, defined as unwanted and/or harmful sound [19, 20], is primarily created by people and the equipment used to maintain life and monitor patients. This is amplified by the poor acoustics of traditional ICU bedspaces with limited sound absorption and blocking, leading to sound transmitting between, and reflecting within, bedspaces.

Noise compounds the effect of artificial lighting and associated disruption of circadian rhythms, contributing to the sleep deficit frequently experienced by ICU patients [2, 3, 7]. This can increase mortality and morbidity, including delirium, psychological disturbances, cognitive problems, impaired immune function, prolonged mechanical ventilation, and development of a catabolic state [2, 3, 6, 9, 10, 16, 17, 21–29]. Poor sleep may therefore prolong length of stay and increase the cost of the healthcare episode [25]. Excessive sound can also impact staff health and contribute to staff error, stress, decreased job satisfaction and motivation, thereby contributing to burnout and turnover at immense personal and economic costs [16, 17, 30, 31].

Numerous solutions to environmental problems have been proposed and tested. Some, including eye masks and ear plugs [32–34], have shown efficacy. However, these solutions are designed to mask or reduce patients’ perceptions of light and noise rather than addressing the underlying problems. Moreover, effectiveness is compromised by reduced compliance with eye masks and ear plugs related to patient discomfort and anxiety associated with sensory deprivation [35–37].

Recognition that environmental improvement is needed underpins authoritative calls for redesign of ICUs to enable holistic and personalised care [38]. Given the current and expected future ICU workforce shortage, and the high rate of staff health concerns, burnout, and turnover (with 67% of ICU nurses intending to leave their jobs in the next 3 years) [39–42], it is essential that the environment is also optimised for staff well-being and retention. However, meaningful redesign has yet to be realised. Given the diversity of contexts, patient cohorts, and models of care internationally, there is unlikely to be an ‘ideal’ or standardised ICU environment applicable across all settings. Rather, efforts to optimise the ICU environment must be shaped to suit context, budget, and importantly, address local problems using bespoke solutions.

To address these issues, we aimed to co-design and implement an improved ICU environment, optimising delivery of care, patient recovery, and staff well-being. Preceding and guiding the ICU co-design, this project used mixed methods to examine the current environment from the perspectives of stakeholders (clinicians, patients, and families). Previous manuscripts have reported qualitative findings from stakeholders’ experiences and views about improvement [43, 44]. Consistent with international literature, stakeholders were concerned with light and sound, identifying various problems. An objective measurement of the environment was subsequently conducted, focussing on acoustics, sound, and light to further inform the redesign process and to establish a baseline for comparison.

## Methods

### Setting

This observational study was undertaken between 2018 and 2022 in an Australian quaternary ICU specialising in cardiothoracic medicine and surgery. The ICU, opened in 2008, comprises 27 beds in three, nine-bed ‘pods’. Twenty-one beds are open-plan, with bedspaces mainly separated by curtains; six beds are single rooms. Twenty bedspaces have windows.

Data were initially collected in three locations selected to represent different types of bedspaces in the ICU: ‘Bedspace 1’—a single room with a south-facing window, ‘Bedspace 2’—an open-plan bedspace furthest from the nurses’ station and unit entrance with a north-facing window, and ‘Bedspace 3’—an open-plan bedspace adjacent to the nurses’ station and ICU entry, without a window. ‘Bedspace 4’—an open-plan bedspace without a window, was subsequently evaluated after it was identified as one of the bedspaces to be redesigned and upgraded.

Based on shift patterns and standard ICU practice, we defined the hours of 22.00–06.00 as ‘night-time’.

### Data collection and analysis

The study was approved by the hospital Human Research Ethics Committee (HREC/18/QPCH/82). Data were collected over several time periods (Table 1).

**Table 1:** Summary of study periods and measures collected

Data/measure	Collected when	Collected where	Collected how	Comments
Light intensity and doors open versus closed	August 2018	Study bedspaces 1, 2, 3	Urbanise IOT wireless sensors	Sensors situated as per hospital regulations and Australian standards
Sound	August 2018	Study bedspace 2	Brüel & Kjær B&K Type 2250-S sound level meter	Microphone placed in the ceiling directly above patients’ head
Sound	For periods in 2018–2020	Study bedspaces 1, 2, 3	SoundEAR 3–300 noise level monitor	Microphone placed in the ceiling directly above patients’ head
Acoustics	January 2019	One ICU ‘pod’	Background noise levels, reverberation time, and acoustic privacy and separation between spaces	Completed when the ICU ‘pod’ was closed due to reduced activity
Alarms	October 2019	All ICU	Philips Patient Information Center (PIC iX) central monitoring system + manual download from ventilators	Monitor and ventilator data only available
Light—horizontal illuminance levels and spectral power distribution	April 2022	Study bedspaces 2 and 4	Konica Minolta Illuminance Meter T-10 & Asensetek Lighting Passport Pro Standard spectroradiometer	Measured at a height of 72 cm from the floor, corresponding with average bed height in the ICU

### Lighting environment

In the first (primary) study period, data were collected over three weeks in August 2018. Illuminance levels (Lux—bedspaces 1, 2, and 3) and door position (open vs closed—bedspace 1) were continuously collected by Urbanise IOT wireless sensors. These sensors communicated with a central gateway (Urbanise IOT 4G Wireless M2M Gateway) placed in the nurses’ station, which transmitted data to the Urbanise IOT remote monitoring platform, where live and historical data could be reviewed and downloaded.

Sensors were placed according to hospital regulations and Australian standards [45], stipulating a minimum separation of 1.5 m between any portable radio frequency communications equipment and patients. The light sensors were therefore placed on the wall of the bedspaces at the height of the bedhead to best correlate with conditions experienced by patients.

Following agreement about which bedspaces to redesign and rebuild, a more detailed assessment of the lighting conditions and quality of lighting was performed in April 2022 in bedspaces 4 (implementation bedspace) and 2 (reference bedspace). The bedspaces shared a similar electric lighting setup, consisting of five ceiling-mounted square luminaires. The different lighting conditions measured for each bedspace are summarised in Table 2.

**Table 2:** Summary of lighting conditions evaluated

Lighting condition	Room	Time measured	Type of light source
A	Bedspace 4	12 PM	Electric lighting only
B	Bedspace 2	12 PM	Daylight only
C	Bedspace 2	12 PM	Daylight and electric lighting
D	Bedspace 2	6 PM	Electric lighting only

Two metrics were used: Horizontal illuminance levels (measured using a calibrated Konica Minolta Illuminance Meter T-10) describing light intensity across the room, and spectral power distribution (SPD—measured using an Asensetek Lighting Passport Pro Standard spectroradiometer), which characterises energy emissions at different wavelengths across the electromagnetic spectrum [46]. The measurement setup (Additional file 1: Fig. S1) was guided by the Australian Standard 1680.1:2006 Appendix B [47]. Both instruments were positioned 72 cm above the floor, corresponding with the average height of beds in the study ICU.

### Acoustic environment

#### Sound

Sound levels were initially measured utilising a Brüel & Kjær B&K Type 2250-S sound level meter placed in bedspace 2. A calibrated microphone was placed in the ceiling above the bedhead, with a 3-m extension cable attaching the microphone to the sound level meter. The device records average sound levels (LAeq 15 min—continuous A-weighted sound pressure level values averaged over 15 min) and peak sound levels (LAFmax—the maximum A-weighted sound level with fast time weighting).

During subsequent data collection, SoundEar 3–300 noise level monitors were deployed in bedspaces 1, 2, and 3. This device records average sound levels as LAeq1min (continuous A-weighted sound pressure level values averaged over 1 min) and peak sound levels as LAFmax. The devices were connected to a calibrated high-fidelity microphone attached to the ceiling as described above.

Acoustic interruption (AI) is the difference between background sound levels and short, high sound spikes. We selected a difference of 17 dBA (A-weighted decibel) between average and peak sound levels as the value for defining AIs as this has previously been shown to cause an arousal from sleep [48].

#### Alarms

Alarm data were collected for 31 days in October 2019. Patient monitor alarm data (number and type) were analysed using the Philips Patient Information Center (PIC iX) central monitoring system. Ventilator alarm logs for the same period were downloaded from ventilators undergoing service between October and December 2019 (n = 7), with average data used to estimate the number of alarms across all ventilators.

#### Acoustics

Acoustics were assessed in January 2019 in an empty ICU ward in three ways. First, background sound levels (dBA) were measured. All doors to the ward were closed minimising external sound intrusion; therefore, any sound present was background sound from mechanical services. Subsequently, reverberation time (RT—the time taken for a loud sound to reduce by 60 dBA (in seconds) and therefore a measure of acoustic absorption) was evaluated through the generation of a short-term high sound level immediately followed by silence. Finally, acoustic privacy and separation between spaces across sealed partitions and doors (a measurement of the amount of external sound intrusion into patients’ bedspaces) was tested by generating a high noise event using a large speaker and white noise.

All data described above were downloaded to Microsoft Excel (version 2208) and analysed descriptively using measures of central tendency, range, and frequency.

## Results

### Lighting environment

**Fig. 1:**
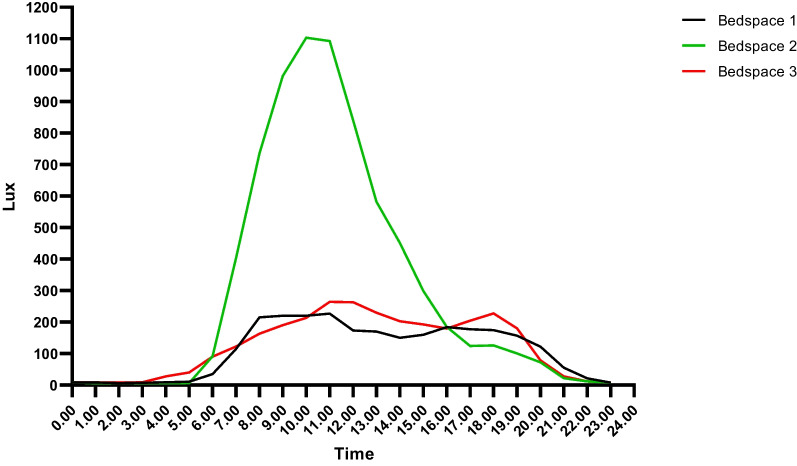
Mean light intensity for bedspaces 1, 2, and 3 across the day (averaged over 3 weeks). Bedspace 1: single room with window, bedspace 2: open-plan bedspace with window, and bedspace 3: open-plan bedspace without a window

All bedspaces showed light intensities well below outdoor illuminance. Light intensity varied between bedspaces and also within bedspaces on different days (Fig. 1 and Additional file 1: Fig. S2). Bedspace 2 (open-plan bedspace with a window) had greater light intensity between 07.00 h and 14.00 h and followed a traditional diurnal lighting pattern. Bedspaces 1 (single room with a window) and 3 (open-plan bedspace without window) showed substantially less diurnal variation with very similar light intensities over the 24-h period. The data showed minimal differences in light intensity for occupied versus unoccupied bedspaces, with the intensity being slightly higher in two of the bedspaces when unoccupied (see Additional file 1: Fig. S3 for an example).

**Fig. 2:**
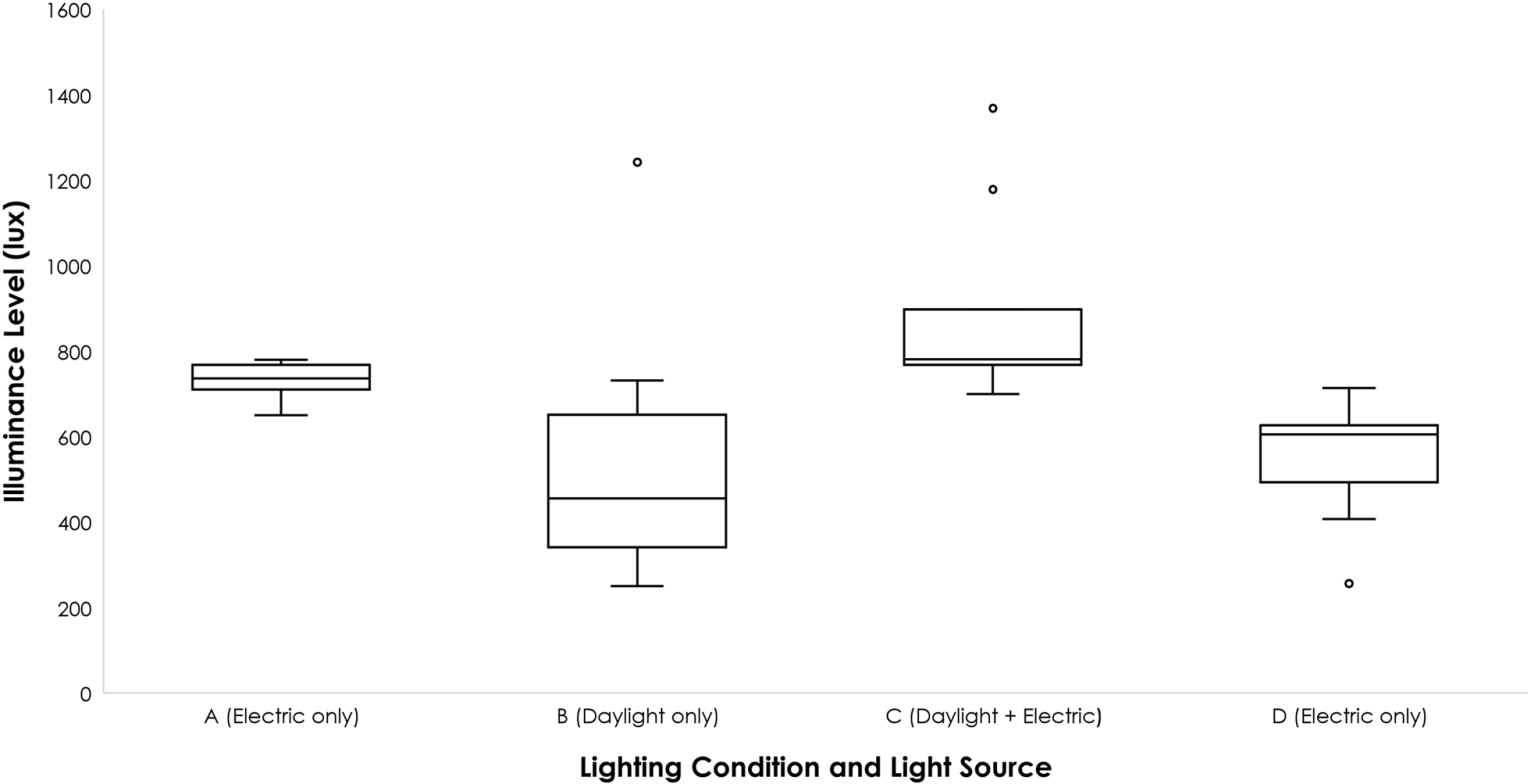
Illuminance levels under different lighting conditions

**Fig. 3:**
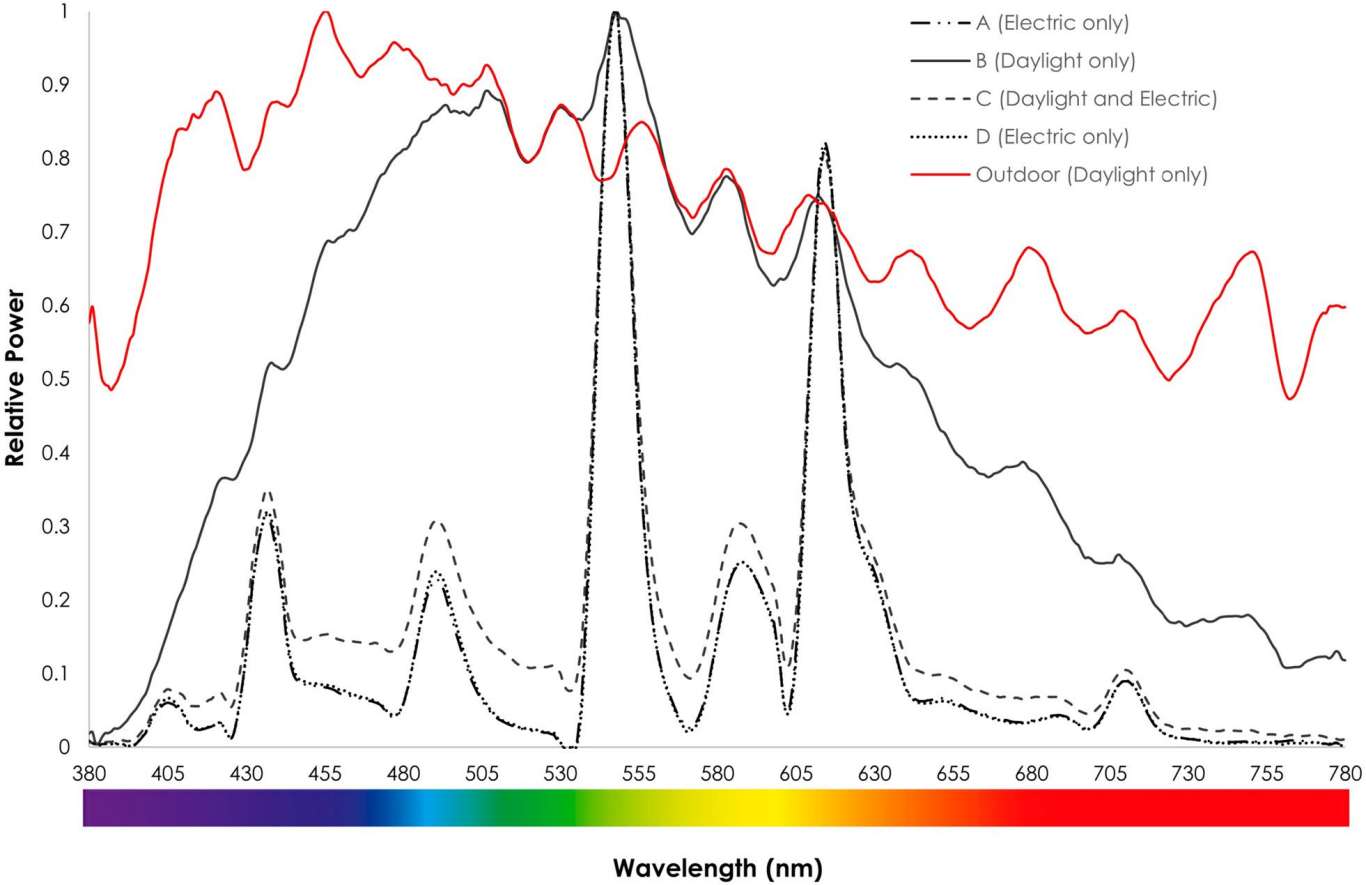
Spectral power distribution under different lighting conditions

A summary of the horizontal illuminance levels across bedspaces 2 (open-plan with window) and 4 (open-plan without window) is presented in Fig. 2; descriptive statistics are shown in Table 3. A summary of compounded SPD values is presented in Fig. 3. Under lighting condition B (daylight only), bedspace 2 (open-plan bedspace with a window) had the broadest spectrum amongst the group, which more closely resembles the lighting spectrum measured outdoors. However, the SPD for that same bedspace shows that the superposition of electric lighting added to the daylight created a combined light that is near identical to the ceiling lights alone.

**Table 3:** Variability of light between different lighting conditions

	A (bedspace 4—electric only)	B (bedspace 2—daylight only)	C (bedspace 2—daylight + electric)	D (bedspace 2—electric only)
Mean (Lux)	728.56	530.89	896.44	540.56
Median (Lux)	737	456	781	605
SD	47.88	311.81	227.03	141.33
Uniformity	0.89	0.47	0.78	0.47

Bedspace 2: open-plan bedspace with window, bedspace 4: open-plan bedspace without window

### Acoustic environment

#### Sound

**Fig. 4:**
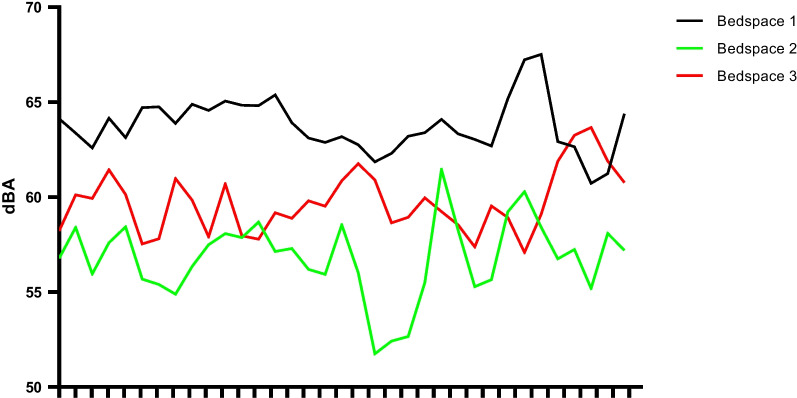
Daily mean sound levels in bedspaces 1, 2, and 3 during the 35-day study period. Bedspace 1: single room, bedspace 2: open-plan bedspace furthest from nurses’ station and unit entrance, and bedspace 3: open-plan bedspace closest to nurses’ station and unit entrance

Sound data are summarised in Fig. 4, Additional file 1: Figs. S4–6, and Table 4. Mean sound levels were 63.75, 56.80, and 59.71 dBA in bedspaces 1 (single room), 2, and 3 (both open-plan), respectively. Diurnal variation was greater in open-plan bedspaces with a difference of 8 dBA between daytime and night-time, compared to approximately 2 dBA for the single room. All bedspaces recorded peak sound levels above 100 dBA, with the highest being 105.6 dBA. The mean number of peak sounds > 80 dBA per hour was 7.74, 3.30, and 6.29 in bedspaces 1, 2, and 3, respectively, with a higher number recorded during the day.

**Table 4:** Summary of sound data for bedspaces 1, 2, and 3. dBA; A-weighted decibel, Range; minimum–maximum, SD; standard deviation

	Bedspace 1	Bedspace 2	Bedspace 3
Mean sound levels [SD, range] (dBA)	63.75 [3.67, 56.40–86.40]	56.80 [6.16, 41.70–83.80]	59.71 [6.07, 40.30–81.30]
Mean sound levels—daytime [SD, range] (dBA)	64.33 [3.61, 56.40–86.40]	59.52 [4.98, 42.50–83.80]	62.48 [4.26, 43.50–81.30]
Mean sound levels—night-time [SD, range] (dBA)	62.58 [3.50, 57.10–79.60]	51.35 [4.46, 41.70–76.40]	54.17 [5.28, 40.30–77.30]
Highest recorded peak max sound level (dBA)	104.0	105.6	102.6
Mean hourly acoustic interruptions > 17 dBA [SD, range] (count)	1.28 [1.72, 0–11]	3.65 [2.66, 0–17]	4.25 [3.19, 0–24]
Mean hourly acoustic interruptions > 17 dBA—daytime [SD, range] (count)	1.53 [1.83, 0–11]	3.41 [2.52, 0–17]	3.45 [2.57, 0–16]
Mean hourly acoustic interruptions > 17 dBA—night [SD, range] (count)	0.77 [1.37, 0–9]	4.13 [2.84, 0–14]	5.85 [3.67, 0–24]
Mean hourly peak counts > 80 dBA [SD, range] (count)	7.74 [8.62, 0–47]	3.30 [4.24, 0–26]	6.29 [6.10, 0–36]
Mean hourly peak counts > 80 dBA—daytime [SD, range] (count)	9.48 [8.39, 0–37]	4.56 [4.58, 0–26]	8.37 [6.07, 0–36]
Mean hourly peak counts > 80 dBA—night-time [SD, range] (count)	4.24 [7.98, 0–47]	0.76 [1.48, 0–11]	2.11 [3.44, 0–24]

Acoustic interruptions: the difference between background sound levels (averaged over a one-minute period) and short, high sound spikes; Mean sound levels: LAeq1min = continuous A-weighted sound pressure level (SPL) values averaged over 1 min; Peak max sound levels: LAFmax = the maximum sound level with 'A' frequency weighting and fast time weighting

Bedspace 1: single room, bedspace 2: open-plan bedspace furthest from nurses’ station and unit entrance, and bedspace 3: open-plan bedspace closest to nurses’ station and unit entrance

#### Acoustics

Acoustic testing results are summarised in Additional file 1: Fig. S7. The RT was 0.7 s in open-plan bedspaces and 0.8 s in single rooms. Background sound varied between 36 and 41 dBA and 36 and 44 dBA, respectively, in the open-plan bedspaces and single rooms.

#### Alarms

**Fig. 5:**
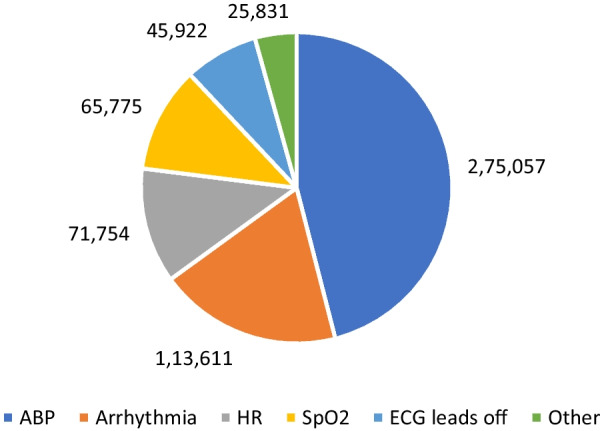
Number of non-actionable monitor alarms from different causes in one month

Audit of patient monitor alarms identified 600,452 alarms over the 31-day study period (14,729 occupied bed hours/average daily bed occupancy = 20). A mean of 40.77 alarms occurred per occupied bed hour (approximately one new monitor alarm per bedspace every 88 s). Of the 600,452 monitor alarms, only 30,023 were actioned (5%), with 46% of non-actionable alarms being arterial blood pressure alarms, 19% were for arrhythmias, 12% heart rate, and 11% SpO_2_ (Fig. 5).

Audit of ventilator alarms estimated 172,061 alarms for the study period, equating to 11.68 alarms per occupied bed hour (one new alarm every 308 s). Combining monitor and ventilator alarms, there was an estimated one new alarm every 69 s per bedspace. No data were available for other bedside equipment.

Door sensors (Additional file 1: Fig. S8) showed that the doors to the single room were kept open most of the time, particularly when the bedspace was occupied.

## Discussion

This study describes the light, sound, and acoustic environment in the study ICU. Consistent with studies of other ICUs, light levels were below, and sound above recommended levels [5, 9, 10, 49, 50]. The alarm burden was especially high. Findings have been applied in redesign efforts and will be used to estimate the effectiveness of proposed solutions. Findings have implications for evaluation and design of ICUs worldwide. Table 5 summarises proposed solutions discussed below to problems identified.

**Table 5:** Summary of the main problems identified and potential design and technological solutions available to address these

	Problem	Solution
Lighting	Loss of circadian entrainment	Circadian lighting
Noise	Alarm burden	Redirect alarms to intended hearer
		Alarm management solutions
Acoustics	Noise pollution from outside bedspace	Single rooms/enclosed space (ensuring doors are closed overnight/during rest periods)
		Optimised sound blocking
	Noise pollution from within the bedspace	Sound masking/replacing unwanted sounds with sounds chosen by patient
		Optimised sound absorption
		Staff education
		Noise loggers with visual warning

### Lighting environment

There are opportunities to improve daytime and night-time light in all bedspace types studied. Optimising lighting and supporting circadian rhythms, ensuring night-time sleep and daytime wakefulness, are critical to best outcomes. However, there is variation in recommendations regarding light levels required to entrain circadian rhythms. One study suggested a light intensity of 1000 lx is required [10], with others recommending several hours of ≥ 2500 lx of blue weighted light [51]. Our finding of < 300 lx during daytime hours in both windowed and windowless bedspaces suggests that the current ICU environment is insufficient to maintain circadian entrainment.

Light intensity was slightly higher when the bedspaces were unoccupied, suggesting that staff/patient factors may influence the lighting environment. Optimising bedspace lighting requires not only optimal lighting (using natural light when available and optimising electrical light patterns to best mimic natural daylight when not) but also needs to address human factors (e.g. staff behaviour). Programmed and automated lighting solutions are available, but staff can override this if not educated on the essential role light plays in entraining circadian rhythms and supporting sleep.

Natural light is an essential part of an optimised ICU environment [7, 12, 22, 52]. However, windows are commonly located behind patients, limiting the natural light reaching the patient’s retina, which is essential for circadian entrainment [53, 54]. Further, our results demonstrate for the first time that traditional ceiling lights override and thus negate the benefit of any available natural light, leaving the effects of windowed and windowless bedspaces to be largely the same. Circadian lighting solutions have been reported to entrain circadian rhythms, reducing the delirium rate, and improving immune function, sleep, cognitive abilities, metabolic function, and productivity, demonstrating the impact of a simple ICU design improvement [24, 51, 53–55]. While this clearly has potential benefits in windowless bedspaces, our data suggest that even bedspaces with windows could benefit from dynamic lighting solutions. However, it has not been demonstrated that dynamic electric lighting can replicate the positive outcomes achieved with natural daylight [51], and there are limited data evaluating the impact of circadian lighting solutions on patient outcomes. More research is required to determine how improved lighting impacts on staff health and performance, and patient experience and outcomes.

### Acoustic environment

Sound levels recorded were similar to other studies [16, 56]. The open-plan bedspace closer to high activity areas like the nurses’ station and ICU entrance had higher sound levels than the bedspace further away. Similarly, the limited difference in sound levels between occupied and unoccupied bedspaces indicates that a lot of the sound is generated outside the bedspace itself and is emanating from other areas of the ICU and adjacent bedspaces in an open-plan unit, highlighting the need for optimised bedspace sound blocking.

All bedspaces recorded multiple counts of peak sound levels above 80 dBA overnight. To enable sleep, sound levels < 40 dBA are recommended [25, 57]. However, the threshold for waking may increase when patients are continually exposed to a noisy environment [58]. Sound variability, AIs, and peak sound levels are therefore more likely to negatively impact sleep than ambient sound, as our brains are better at ignoring continuous sound than sudden changes [23, 49, 59]. This occurred frequently and was more common at night (when the ambient sound was lower).

A key finding was the large number of alarms recorded. We found approximately 1,250 monitor or ventilator alarms per bedspace per day, compared with previous studies reporting 100–771 daily [60–62]. One new alarm commenced every 69 s in each bedspace, delivered adjacent to the patient’s head (even though bedside alarms are there to notify staff, not patients) [2, 63]. If we include all other bedside equipment, the actual frequency of alarms in the bedspace would be even greater. With most bedspaces being open-plan, alarms generated in nearby bedspaces are likely to also be audible. Further, not all alarms are accurate or critical. Studies suggest that almost 90% of alarms in ICU are false or non-actionable [2, 16], meaning detrimental effects caused by these alarms are not balanced with a corresponding benefit. Ninety-five per cent of alarms in this study were not actioned.

Single rooms have theoretically improved the situation by ensuring less external sound is transmitted into the bedspace. However, this is dependent on sufficient sound blocking and an optimised acoustic environment to absorb internally created sounds. Similar to our findings, studies have shown that sound levels can be higher in single rooms than in open-plan bedspaces [63]. This may be because patients in single rooms are more acutely unwell and require more supportive equipment, staff behaving differently when in single rooms, or because of more family members being present (e.g. during palliation). The single rooms in the study unit had no acoustic absorption; therefore, any sound created could reflect and reverberate around the room. Also, doors were kept open most of the time, negating their ability to block sound. Careful selection of building materials and an improved layout could assist with addressing these issues.

In our study, we found RTs above the recommended levels of 0.6 s [64], displaying poor acoustic absorption and contributing to the high sound levels. Similarly, the Australian standards for building interiors recommend a background sound level range of 40 to 45 dBA [64]; however, some of our bedspaces only recorded a background sound level of 36 dBA. A higher background sound level is recommended as it is a contributing factor to maximising acoustic privacy and masking peak sound levels.

Some solutions to address excessive sound levels are easy to implement and inexpensive. Studies have shown that increasing awareness of noise through education and/or by placing noise loggers with a visual display showing sound levels are effective in reducing noise [17, 65, 66]. Some modern patient monitors include smart alarms, utilising trends in measurements rather than threshold alarms, thereby reducing the number of alarms. Quiet and silent alarm solutions, sending alarms directly to the caregiver via handheld devices, are becoming available but not commonly used yet. Excessive sound can be masked, or replaced by more pleasant sounds, using technology rather than relying on physical barriers or ear plugs. Sound masking (the use of white noise) has shown positive impacts when trialled in ICUs [67–69]. This assists with masking unwanted or unpleasant sounds. Also, by raising the background sound levels, sound masking reduces the gap between peak and background sound levels. When implemented together with relevant strategies to reduce the loudness of peak sound levels, this can decrease the number of AIs and therefore sleep disruptions experienced by patients. There are also various ways to mask noise using sounds chosen by patients. Portable speakers or beds with in-built wireless speakers allow individualised music or nature sounds to be played directly to the patient.

As has been demonstrated by this and other studies, reasons for excessive sound levels in ICU are multifactorial, and the current ICU bedspace design suboptimal for creating a good acoustic environment. Any effective solution to address this problem will therefore need to address multiple factors simultaneously. This includes addressing the location and number of alarms being created, while utilising various strategies to control or mask sounds that are disrupting patients’ sleep and recovery. Ensuring optimal sound absorption and blocking within and between bedspaces will need to be addressed to optimise the acoustic environment, minimising reverberation while ensuring infection control is not compromised. A lot of the excessive sounds perceived as noise by patients in ICU is preventable, and the prevention of these should be the focus of future studies, together with studies on how the unpreventable sources of noise can be moved away from patients and better masked to limit the negative impact.

### Limitations

There are several limitations of this study that constrain generalisability of data. Firstly, this is a single-centre observational study; therefore, implications of sounds and light are based on previous studies, and findings specific to the study site, staffing ratio, and context. However, reported findings are similar to previous studies.

Secondly, staff may have altered ‘normal’ behaviour if sensors and microphones were observed. While staff were not explicitly told about the purpose of the study and the environmental monitoring, the sensors and microphones were visible. It is likely that some modification of staff behaviour occurred. To mitigate the impact, sensors and monitors were installed one month prior to the commencement of data collection, with the assumption that staff would get used to their presence and revert to normal behaviours before data collection commenced.

Thirdly, it is unlikely that the data precisely reflect the environment as experienced by patients. Hospital regulations and standards stipulated that environmental sensors used in ICU were required to be positioned at least 1.5 m away from patients due to concerns about potential electromagnetic interference and impact on patient monitoring. Sensors therefore had to be placed in the most suitable location that was practically possible, either on the wall at the level of the patients’ head, or in the ceiling directly above the patients. Therefore, the location of the sensors was not optimal. For instance, the light sensors were likely to measure the lighting levels available in their location (and may have had direct sunlight hitting them at times during the day) rather than the lighting levels available at the level of the patients’ eyes (who were all facing away from any windows). Similarly, decibel levels recorded are the sound levels in the ceiling (where the microphone was situated), not the level at the patient’s ears. Depending on the type and origin of the sound, this may have had a large impact on the sound levels recorded. For instance, direct sounds such as ventilator and monitor alarms originated closer to the patients’ head (60 cm and 90 cm, respectively) than the microphone (160 cm and 100 cm, respectively), therefore being perceived as louder for the patient than the sound levels recorded. Conversely, transmitted and reflected sounds may be more likely to be directed towards the ceiling where there are no soft surfaces to help absorb or block some of the sound waves.

Finally, most of the data were collected over one period in winter, which corresponds with a busy time of year traditionally in the study ICU. This may have impacted on some of the data collected. However, we collected lighting data over a 12-month period and confirmed there was no difference across seasons. Similarly, the number of patients admitted during the period of data collection was around the average for the unit. Therefore, it is unlikely that sound levels recorded were significantly different to other times of the year.

## Conclusion

This study has described the sensory environment of a large quaternary ICU in preparation for an environmental upgrade and ICU bedspace redesign. The study corroborates previous findings and confirmed that the lighting and acoustic environments in the study ICU were suboptimal and potentially contributing to adverse patient outcomes. Future ICU design should consider various strategies to ensure that the lighting and acoustic environment in ICUs are optimised to suit the needs of all end-users, including patients, families, and staff. Future studies are needed to determine whether an optimised ICU environment positively impacts patient outcomes.

### Supplementary Information

**Additional file 1**. Lighting measurement grid for each bedspace (bedspace 4 (without window) on the left and bedspace 2 (with window) on the right).

## Data Availability

The datasets are available from the corresponding author on reasonable request.

## Abbreviations

AI: Acoustic interruption
dBA: A-weighted decibel
ICU: Intensive care unit
LAeq1min: Continuous A-weighted sound pressure level values averaged over 1 min
LAeq 15 min: Continuous A-weighted sound pressure level values averaged over 15 min
LAFmax: Maximum A-weighted sound level with fast time weighting
PIC iX: Philips Patient Information Center
RT: Reverberation time
SPD: Spectral power distribution
