# Supplementary material for: Evaluation of the sensory environment in a large tertiary ICU
Source: Crit Care. 2023 Nov 27;27:461. doi: 10.1186/s13054-023-04744-8 (PMC10683296; doi:10.1186/s13054-023-04744-8)
Supplement: Supplementary file 1 — Additional file 1. Lighting measurement grid for each bedspace (bedspace 4 (without window) on the left and bedspace 2 (with window) on the right). [file 13054_2023_4744_MOESM1_ESM.docx]

Supplementary Figure 1


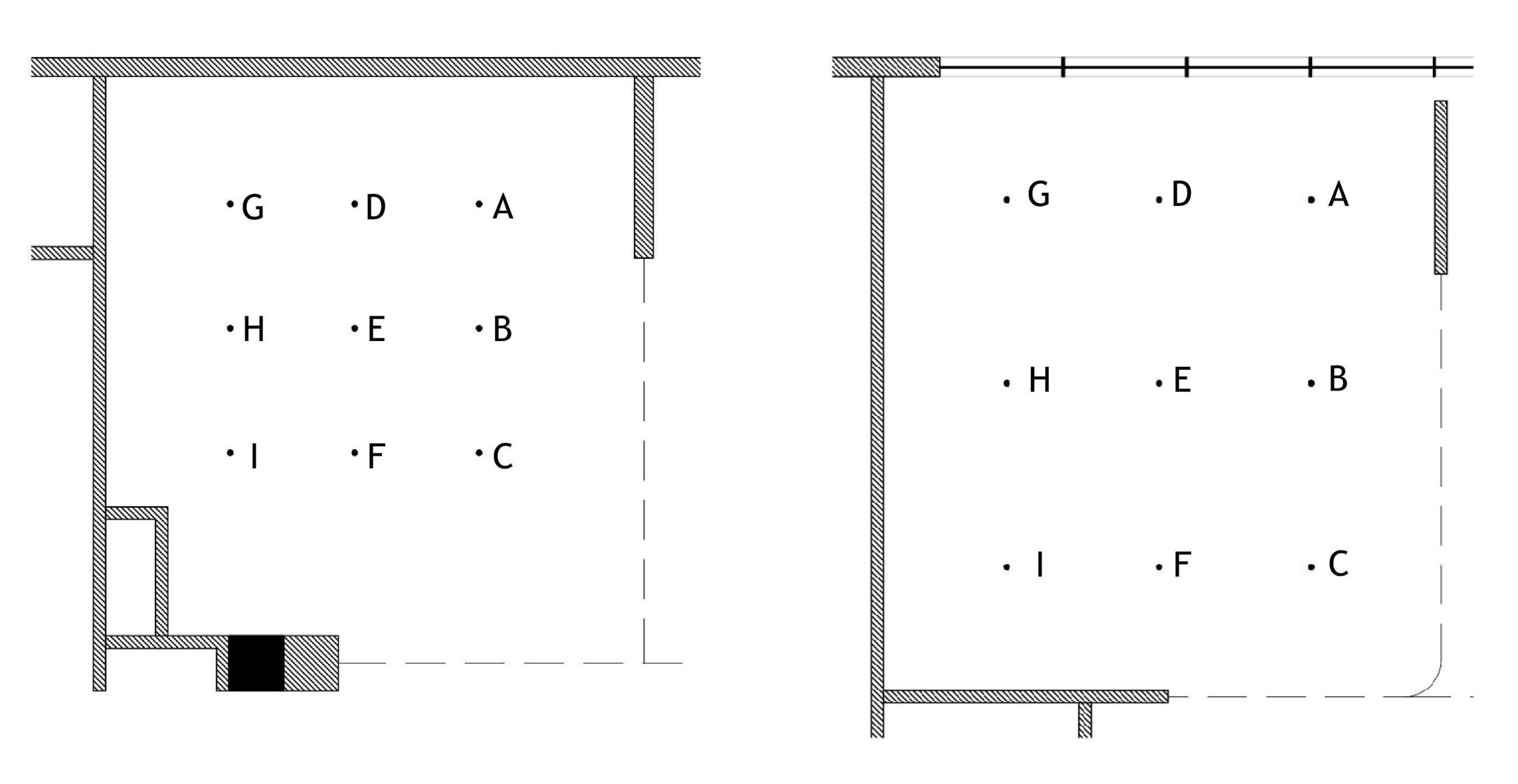


*Supplementary figure 1 – lighting measurement grid for each bedspace (bedspace 4 (without window) on the left and bedspace 2 (with window) on the right)*

Supplementary Figure 2

*Supplementary figure 2 – mean light intensity in bedspace 1 (when occupied) as an example comparing days of high and low light intensity against the mean light intensity across 24 hours*

Supplementary Figure 3

*Supplementary figure 3 – mean light intensity in bedspace 3 across 24 hours as an example comparing occupied versus unoccupied periods*

Supplementary Figure 4

*Supplementary figure 4 – mean sound levels across 24 hours in bedspaces 1, 2, and 3 during the study period.*

Supplementary Figure 5

*Supplementary figure 5 – mean count of peak sound levels >80 dBA across 24 hours in bedspaces 1, 2, and 3 during the study period*

Supplementary Figure 6

*Supplementary figure 6 – mean acoustic interruptions >17dBA across 24 hours in bedspaces 1, 2, and 3 during the study period*

Supplementary Figure 7


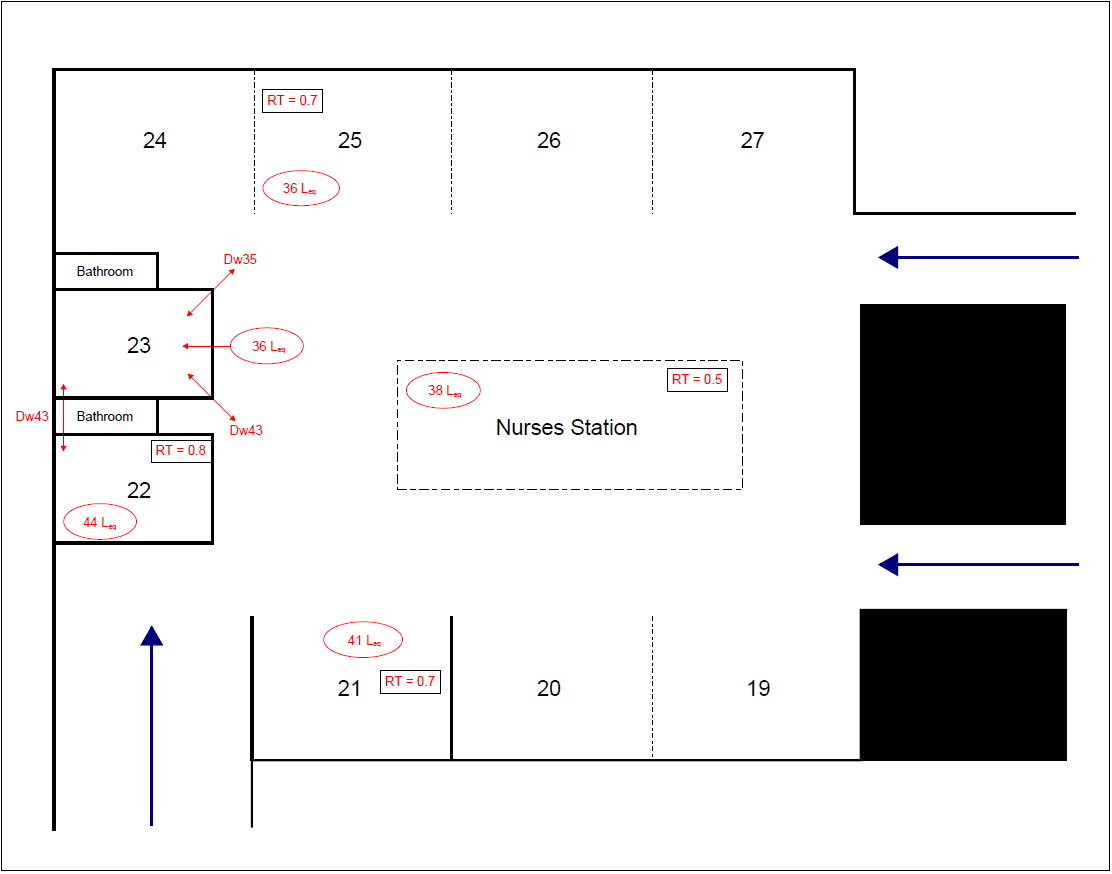


*Supplementary figure 7 – results of acoustic evaluation of one of the study ICU wards*

*dBA: A weighted decibels (weighted according to the weighting curves to approximate the way the human ear hears); Dw: weighted level difference (measure of noise transmission loss across partitions); Leq: equivalent continuous sound level (measure of background noise level in the space); RT: reverberation time (the time taken for a loud noise to reduce by 60 dBA (in seconds))*

Supplementary Figure 8

*Supplementary appendix 8 – door between single room (bedspace 1) and the nurses’ station open vs closed across 24 hours during the study period – comparing occupied vs unoccupied periods*

*The sensor detects whether the door is open or closed. 0% indicates the door is continuously kept open in the 60-minute period, 100% indicates it’s continuously closed*
